# A GIS Analysis of the Relationship between Sinkholes, Dry-Well Complaints and Groundwater Pumping for Frost-Freeze Protection of Winter Strawberry Production in Florida

**DOI:** 10.1371/journal.pone.0053832

**Published:** 2013-01-11

**Authors:** Mark D. Aurit, Robert O. Peterson, Justine I. Blanford

**Affiliations:** 1 Tampa Bay Water, Clearwater, Florida, United States of America; 2 Water Resources Bureau, Southwest Florida Water Management District, Brooksville, Florida, United States of America; 3 Department of Geography, GeoVISTA Center and Dutton e-Education Institute, Penn State University, University Park, Pennsylvania, United States of America; United States Department of Agriculture, United States of America

## Abstract

Florida is riddled with sinkholes due to its karst topography. Sometimes these sinkholes can cause extensive damage to infrastructure and homes. It has been suggested that agricultural practices, such as sprinkler irrigation methods used to protect crops, can increase the development of sinkholes, particularly when temperatures drop below freezing, causing groundwater levels to drop quickly during groundwater pumping. In the strawberry growing region, Dover/Plant City, Florida, the effects have caused water shortages resulting in dry- wells and ground subsidence through the development of sinkholes that can be costly to maintain and repair. In this study, we look at how frost-freeze events have affected West Central Florida over the past 25 years with detailed comparisons made between two cold-years (with severe frost-freeze events) and a warm year (no frost-freeze events). We analyzed the spatial and temporal correlation between strawberry farming freeze protection practices and the development of sinkholes/dry well complaints, and assessed the economic impact of such events from a water management perspective by evaluating the cost of repairing and drilling new wells and how these compared with using alternative crop-protection methods. We found that the spatial distribution of sinkholes was non-random during both frost-freeze events. A strong correlation between sinkhole occurrence and water extraction and minimum temperatures was found. Furthermore as temperatures fall below 41°F and water levels decrease by more than 20 ft, the number of sinkholes increase greatly (N >10). At this time alternative protection methods such as freeze-cloth are cost prohibitive in comparison to repairing dry wells. In conclusion, the findings from this study are applicable in other agricultural areas and can be used to develop comprehensive water management plans in areas where the abstraction of large quantities of water occur.

## Introduction

The US is the world’s largest producer of strawberries [1]. Although, California is the largest producer of strawberries in the US, Florida is the largest producer of winter strawberries due to the mild winter climate [1]. The main growing area in Florida is in the Dover/Plant City Area of Hillsborough County, east of Tampa, where approximately 5800 acres are harvested annually [2].

In Florida, strawberries are planted in November and peak during March (see [3] for timeline). Although winter temperatures are mild ranging between 60.5°F and 74.6°F [4], strawberries and other horticultural crops are sometimes exposed to frost-freeze events when temperatures drop below freezing for short periods of time (see [5]). During these conditions, farmers use either active or passive methods to protect fruit crops [6]. Active methods can include the use of heaters, wind machines, sprinklers, surface irrigation, foam insulation, or a combination of each [7]. Passive methods can include site selection, plant selection, plant nutritional management, plant covers, irrigation, and soil covers [8]. In Florida, farmers use sprinkler irrigation, soil banking and/or cover crop methods [9] to protect crops during frost-freeze events. Of these methods, the sprinkler irrigation method is the most widely used, not only in Florida but in other locations where crops may be damaged by frost-freeze events (e.g. Canada [10]; Louisiana [11]), because it is relatively easy to administer and cost-effective (e.g. $4 vs $93 oil heaters [12]). Essentially, water is sprayed onto the plants, forming a layer of ice on the plant or fruit crop that protects the plants from the cold air temperatures. However, a disadvantage is large quantities of water are pumped from underground aquifers in a relatively short period of time, which can affect water quality, cause water shortages and sinkholes [13]–[15]. Since 1985, population and farm acreage have increased in this area resulting in higher demands of water both by farmers and home-owners, which has the potential to amplify water shortage issues and accelerate sinkhole development [16].

Florida is mainly composed of limestone and is prone to sinkhole development through dissolution by water [17]. Since 1950 there have been a total of 3,109 sinkholes reported to the Florida Geological Survey (FGS) with the greatest number occurring in 1980’s and 2010 [18] (see Figure S1). Although the number of reported sinkholes has declined over recent years, as compared to the 1980’s when reporting was highest due to high ground-water abstraction and concurrent drought conditions [18], numbers increased dramatically during the winter of 2009–2010 (*N* = 179 for 2 months). Sinkholes not only can threaten the welfare of the population, but cause extensive damage [16], [19]–[22]. For example in Florida, during January 1977, groundwater was pumped for six consecutive nights from the Floridan Aquifer to protect strawberry crops resulting in a 9.51 foot reduction of the water level and an increase in the number of new sinkholes [23]. Some of which were large enough to cause damage to property and infrastructure. In 1981 in Orange County, FL, a massive sinkhole measuring 320 feet wide by 90 feet deep destroyed multiple homes, businesses and swimming pools [20], [24], [25]. In 1985, 27 sinkholes, between 2 to 75 ft in diameter, were induced during a three day freeze in Hillsborough County, Florida during January when well water levels dropped 17 ft [14]. In 1994, a 15-story sinkhole opened beneath an 80-million-ton gypsum stack pile (toxic industrial waste) at the IMC-Agrico’s New Wales Plant in Polk County, FL [17], [26]. More recently, again in Polk County FL, a large sinkhole was discovered during a routine inspection of an inactive phosphogypsum disposal stack which measured 160 feet in diameter and extended 200 feet below the surface [27]. Recently, in 2010, during a two month period (January and February) more than 140 sinkholes occurred in the Dover/Plant City Area in Hillsborough County, FL, where water was used to protect strawberries [28]. The damage during this event resulted in the destruction of homes and roads with estimated costs of nearly $1 million [29].

Although studies have investigated the linkage between sinkhole development and ground water pumping in Florida, the association between this and strawberry farmers has only been analyzed for specific years. For example, in 1973, Watson and Company showed that while pumping water from an aquifer several sinkholes developed in close proximity to where the pumping occurred both during and after the testing exercise [30]. Using this report, Hall and Metcalfe (1978) [23] suggested that the development of sinkholes, as a result of a freeze event when temperatures dropped to 25°F on January 17^th^, 1977 for 6 days was the cause of 22 sinkholes when water was extracted for the protection of strawberries. Bengtsson [14] further examined changes in water level, in close proximity to strawberry farms, and the development of sinkholes during the frost-freeze event of 1985. His findings suggest that there is strong evidence that sinkholes may be the result of intense irrigation to protect crops. A limitation of the aforementioned study is that only a single event was examined and Bengtsson [14] estimated the minimum water level during the 1985 frost-freeze event.

In this study, we look at how frost-freeze events have affected West Central Florida over the past 25 years with detailed comparisons made between a cold-year (with severe frost-freeze events) and a warm year (no frost-freeze events). Geographic Information Systems (GIS) was used to examine the spatial and temporal relationship of frost-freeze events to the development of sinkholes/dry well complaints in the strawberry farming region of Florida. In this region, abstraction of water, through the use of sprinklers can result in extensive damage through the development of sinkholes and dry-wells. We also examined the economic impact of frost/freeze events from a water management perspective by evaluating the costs associated with repairing and drilling new wells compared with using alternative non-water based crop-protection methods.

## Methods

To analyze the link between ground water pumping during frost-freeze events and sinkhole development, the occurrence of sinkholes over a 25-year time period was investigated. Three specific years were selected to analyze. These include two years with severe-frost-freeze (1985, 2010) events and one year free of frost-freeze events (2007).

Damage to tender crops such as strawberries occur when temperatures range between 32°F (0°C) and 22°F (−1°C) [10], therefore for the purpose of this study, a frost-freeze event was defined to occur when temperatures fall to 32°F (0°C) and below. Minimum daily temperatures were used to identify frost-freeze events and were obtained from NOAA [31]. Daily minimum temperature data between December 1 and January 31st on a yearly basis from 1985 through 2010 were obtained from NOAA’s 11 stations in the Tampa Bay Area [31]. Although stations were within and around the Dover/Plant City study area, the Tampa International Airport temperature station data was used as it had the most consistently reported dataset for the 25 year timeframe. However, temperature data for the remaining 11 stations were used when analyzing sinkhole occurrence during 1985, 2007 and 2010.

Years absent of frost-freeze events were identified by averaging daily minimum temperatures for each winter (December through January) for each year. A warm winter was considered to occur when no temperatures fell below 32°F and the mean winter average was above the 25-year average. Of the years investigated, 2007 satisfied the aforementioned criteria and was therefore selected to represent a warm year for this analysis. The two years with severe frost-freeze events include the recent winter of 2010 during which some of the coldest temperatures were experienced in 25 years and 1985. Although the linkage between strawberry fields and water extraction has previously been analyzed for 1985 by Bengtsson [14], it was included in this study as a comparison and the data was reanalyzed.

During a frost-freeze event, strawberry farmers pump large volumes of water to protect their crops from damage. To understand the impact of using this protection method, the occurrence of sinkholes and dry well complaints with minimum temperatures and water levels were analyzed in close proximity to strawberry farms. Sinkhole data is collected by the Florida Department of Environmental Protection (FDEP) - Florida Geological Survey (FGS) to manage, protect and develop Florida’s natural resources and was obtained from the Florida Geological Survey [18]. This dataset contains the location represented in latitude and longitude of the subsidence incidents and the date each incident was reported. The reports made to the FGS do not provide the cause of the incident, nor have the reports been verified or field checked by a professional geologist. Though the database has true sinkholes, the majority are subsidence incidents. For the purposes of this study all subsidence incidents have been identified as “sinkholes”. Although, this dataset may have limitations associated with diligence in reporting of historical data [32] it has previously been used in other analyses (see [17], [32]) and is the most comprehensive dataset available at this time.

Population data was obtained by census block group data for four decades (1980, 1990, 2000, and 2010) for areas in and around the study area from the Florida Geographic Data Library (http://www.fgdl.org/metadataexplorer/explorer.jsp). Locations of strawberry fields were obtained from SWFWMD [33] and show the location of strawberry fields in the Plant City/Dover Area for 1984, 2007 and 2010. Although, the frost-freeze event for 1985 was analyzed, the strawberry field information used in this study was only available for 1984 and was therefore used to represent the strawberry field locations for 1985. Dry-well complaints data were acquired from Tampa Bay Waters (TBW) Mitigation Database. Each dry well complaint record is geo-referenced and includes latitude, longitude, and associated costs. SWFWMD also recorded 688 dry well complaints, however the data was provided as a total number for the entire freeze event not on a daily basis level, which was needed for the temporal analysis, therefore it was only used in the spatial analysis portion of the study.

Two types of water data were used in this study. Daily water levels (in feet) were obtained from Tampa Bay Water for water monitoring sites in and around the study area. Three monitoring sites contained sufficient data over the 25 year period include: ROMP DV-1 SUWANNEE, ROMP DV-1 AVON PARK, and WE-BUD-BD-18FL. Data for an 8-week period (December 1st through January 31st) were extracted from these sites for each year between 1985 and 2010, with the exception of 1991. For unknown reasons, these monitoring sites did not capture data for 1991. This data was used to examine the relationship between changes in water levels, number of sinkholes, and drywell complaints. In addition, groundwater use permit volumes for crop protection purposes were obtained from SWFWMD [33]. These attributes include, latitude, longitude and daily maximum allowable volume of water that farm is permitted to use during a frost-freeze event (gallons per day (gpd)).

### Analysis

To analyze the spatial distribution of sinkholes/dry-well complaints and their proximity to strawberry fields in the Plant City/Dover Area a variety of spatial analysis tools available in ArcGIS 10 (ESRI, Inc., Redlands, CA) were utilized. Point pattern analysis methods that have successfully been used to analyze patterns of crime (see [34] for overview) in urban areas (eg. [35], [36])), forest environments [37]) and the spatial distribution of 911 calls [38] were utilized. These methods were useful in highlighting key areas where certain types of crime were clustered (e.g. Kernel Density Estimation (KDE), Average Nearest Neighbor (NNI)). Kernel density estimates were used to create surfaces for sinkholes and dry well complaints to easily identify areas where a large number of incidents were concentrated in the study area. The NNI method was used to identify clustering of sinkholes and drywell complaints by examining conformance with the Independent Random Pattern (IRP) by calculating a ratio (R) of the observed mean nearest neighbor distance to the expected mean nearest neighbor distance of sinkholes and dry well complaints. This method calculates a nearest neighbor index based on the average distance from each point feature to its nearest neighboring point feature [39]. Clustering occurs when values of R<1 [40].

Distance to strawberry farms was calculated to the polygon centroid from each sinkhole and dry-well complaint point. The relationship between the numbers of sinkholes, dry well complaints, minimum temperature and change in water level were tested using Spearman’s rank correlation.

### Economic Assessment

In the Tampa Bay Area, a major push is being made to create incentives/cost sharing programs to encourage farmers to use alternative protection methods. During 2010, SWFWMD implemented a Facilitating Agricultural Resource Management Systems (FARMS) to help cost share alternative protection methods for strawberry farmers in Dover/Plant City [41]. SWFWMD covers 75% of the cost farmers will incur for purchasing freeze-cloth to cover the strawberry plants [13]. To understand the economic impact of frost-freeze events in the areas surrounding the strawberry farms, we investigated the cost associated with the maintenance, repair and/or installation of new wells in comparison to using freeze-cloth (a non-water based protection method).

A cost analysis was completed by calculating the total cost of the freeze-cloth that would have been needed during 2010 and compared the total costs recorded in the TBW dry well complaint cost database. To do this we calculated the total cost of freeze-cloth that would be required for each strawberry field. During the winter of 2010 the cost of the freeze-cloth per acre was $2,400, which includes average labor costs of $300 per acre [42]. The area of each strawberry field was estimated from the strawberry farm dataset, previously described, and used to calculate the total cost of the freeze-cloth that would be required using the price above. The total cost associated with reported dry well complaints were extracted from the TBW dry well complaint cost database on an individual report basis between December 1^st^ and January 31^st^ for 2007 and 2010 and the totals calculated. Data was not available for 1985 and was therefore not analyzed in this study.

## Results

Since 1985, in the Dover/Plant City study area, there has been considerable growth in both population and strawberry farming. Population has increased by 257% in 30 years from 91,000 to 234,000 people with the greatest increase occurring between 1980 and 1990 (84% increase from 91,000 to 167,000). In conjunction with this growth, the acreage of strawberry fields has also increased by 389% from approximately 2,800 acres to 11,000 acres.

### Spatial and Temporal Analysis of Sinkholes and Dry Well Complaints

**Figure 1:**
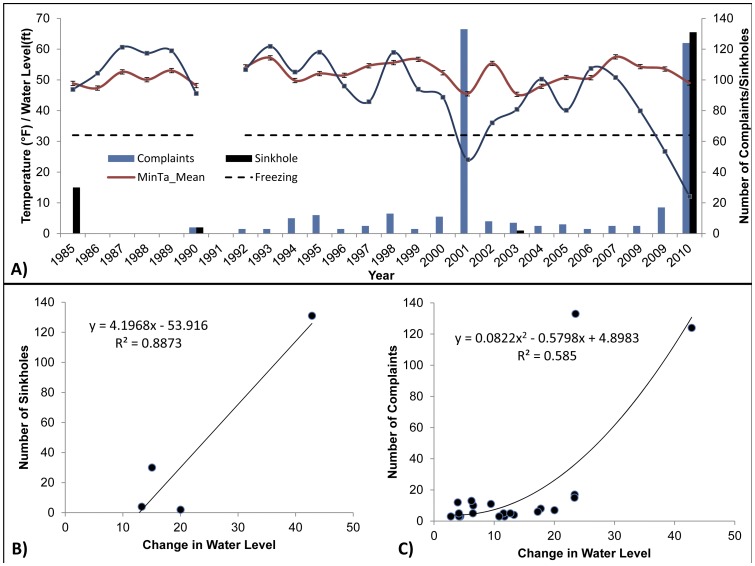
Graph illustrating the occurrence of sinkholes and dry-well complaints between 1985 and 2010 in relation to mean minimum temperature (°F), water level (ft) and changes in water level.

Between 1985 and 2010 the largest number of sinkholes were recorded during the winter of 2010 (N = 131) with the second largest number of sinkholes recorded in 1985 (N = 30) (Figure 1A). Two additional years (1990 and 2003) also had sinkhole incidences, but these were low (N = 2 to 4) in comparison to the aforementioned years. Since 1990, dry-well complaints have been recorded during most years with the highest number of complaints occurring during 2001 and 2010.

Minimum temperatures fluctuated between 50°F and 55°F with the coldest temperatures occurring during 1986, 2001, 2003 and 2010 when average minimum temperatures were below 50°F, 12°F below the average for this time of year (Figure 1). Water levels also varied during this time period with peaks occurring when levels are close to 60 ft and dropping as low as 14 ft during 2010. Water levels dropped for 3 consecutive years reaching a low in 2001 (which coincided with the highest number of recorded dry well complaints). Prior to 2010, water levels also exhibited a similar pattern to 2001, where levels during the December and January were low for 4 consecutive years. Our initial analysis of sinkholes and dry well complaints in relation to minimum temperature and water levels over a 25 year period from 1985 to 2010 clearly show that when water levels fall below 27 feet the number of sinkholes and dry well complaints increase (Figure 1A). A positive relationship was found between number of sinkholes and dry well complaints and changes in water level (Figure 1A and 1B).

**Figure 2:**
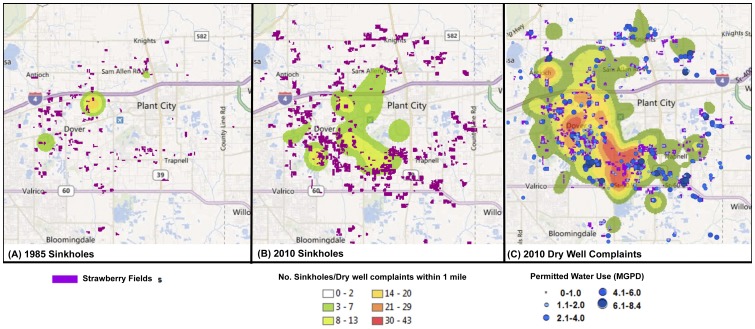
Maps illustrating the density of (A) sinkholes in 1985, (B) sinkholes in 2010 and (C) dry well complaints in 2010 in relation to distribution of strawberry farms and groundwater use permits rates (MGPD).

**Figure 3:**
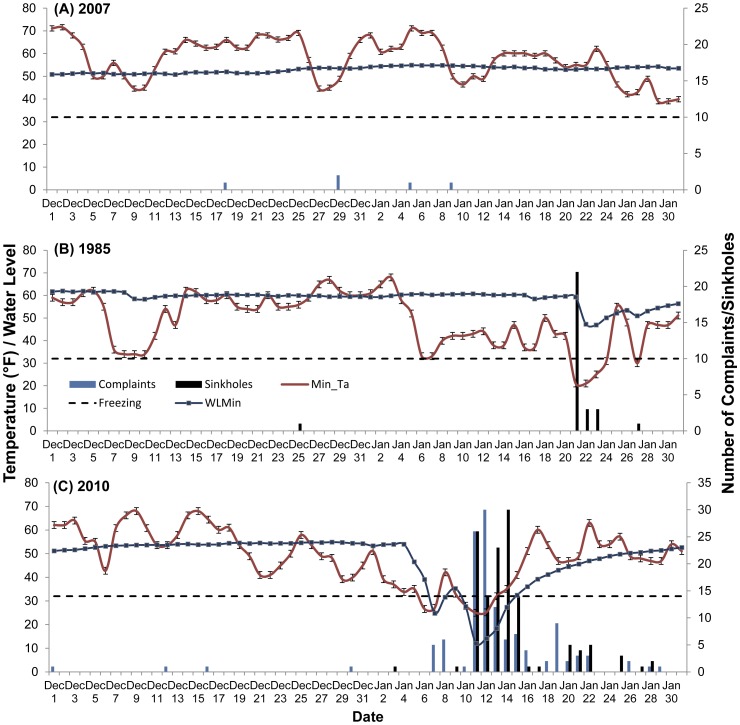
Summary of water level (+/− SE) (WLMin), minimum temperature (°F)(+/− SE)(Min_Ta), and the number of sinkholes and dry-well complaints recorded during December 1^st^ through to January 31^st^ for (A) 2007, (B) 1985 and (C) 2010.

The spatial (Figure 2) and temporal (Figure 3) distribution of sinkholes and dry well complaints in the strawberry growing region of Dover/Plant City were studied for three individual years that include 1985, 2007 and 2010. For this study, the majority of sinkholes and dry well complaints were found to occur within close proximity to the strawberry farms. The majority of the sinkholes occurred within a ¼ mile range of these farms (63% (1985), 45% (2010)), additional sinkholes were recorded up to ½ mile from strawberry fields (0 (1985), 72% (2010)). A similar pattern was observed with dry well complaints with 40% (2007) and 78% (2010) of these occurring within ½ mile of strawberry fields and the majority occurring within a ¼ mile (40% in 2007 and 41% in 2010). During the non-frost-freeze year (December 1, 2006–January 31, 2007) 5 dry well complaints were recorded (Figure 3A) with an apparent random distribution (*NNI = 0.82, P>0.01*). No clear correlation was found between dry well complaints and minimum temperature (*n = 45*, *r* = 0.110, *P*<0.05) and change in water level (*n = 4, r = 0.123, P<0.05*).

Clustering of spatial and temporal occurrence of sinkholes was found during frost-freeze events in 1985 and 2010, and dry well complaints in 2010 (Figure 1–3). Between December 1st, 1984 and January 30, 1985 the incidence of sinkhole and dry well complaints are illustrated in Figures 2A and 3B. NNI results indicated that sinkholes were clustered (*NNI = 0.11, P<0.01*) (Table 1). In 1985 temperatures fell below freezing for a total of 4 days reaching a low of 21°F. During this time water levels dropped 20% (59 ft to 47 ft). A total of 22 sinkholes were reported with the majority being reported over 4 days (Figure 3B). No correlation was found to exist between the number of sinkholes and a change in water level (*n = 5, r = 0.276, P<0.05*).

**Table 1:** Nearest Neighbor analysis test statistics for sinkholes and dry-well complaints during 1985, 2007 and 2010.

Year	Number	Observed Mean Distance (meters)	Expected Mean Distance (meters)	Nearest Neighbor Index	Z-Score	P-value	Clustering
*Sinkholes*							
1985	30	253.88	2362.91	0.11	−9.35	0	Clustered
2010	131	391.75	1126.47	0.03	−14.34	0	Clustered
*Dry Well Complaints*							
2007	5	4730.33	5787.92	0.82	−0.78	0.43	Random
2010	806	205.42	1490.73	0.14	−47	0	Clustered

In the winter of 2010 (December 1, 2009–January 30, 2010) the spatial and temporal distribution of sinkhole and dry well complaints are illustrated in Figures 2B,2C and 3C. Similar to 1985, spatial clustering of sinkholes (*NNI = 0.034, P<0.01*) and dry well complaints (*NNI = 0.14, P<0.01*) were also found (Table 1). During 2010, highest density of dry well complaints and sinkholes were found in close proximity to larger strawberry fields (Figure 2B, 2C).

Temperatures fell below freezing twice for a total of 7 days reaching a low of 25°F (Figure 3C). During these two days water levels dropped over 50% (54 ft to 25 ft) with 11 dry well complaints reported. Following the second cold spell, when temperatures remained low for 5 consecutive days, water levels dropped 78%. A total of 130 sinkholes were reported with the majority being reported over 20 days after the end of the cold temperatures. Over the same 20 day period, 118 dry well complaints were reported to Tampa Bay Water and 688 were reported to SWFWMD for a combined total of 806.

**Figure 4:**
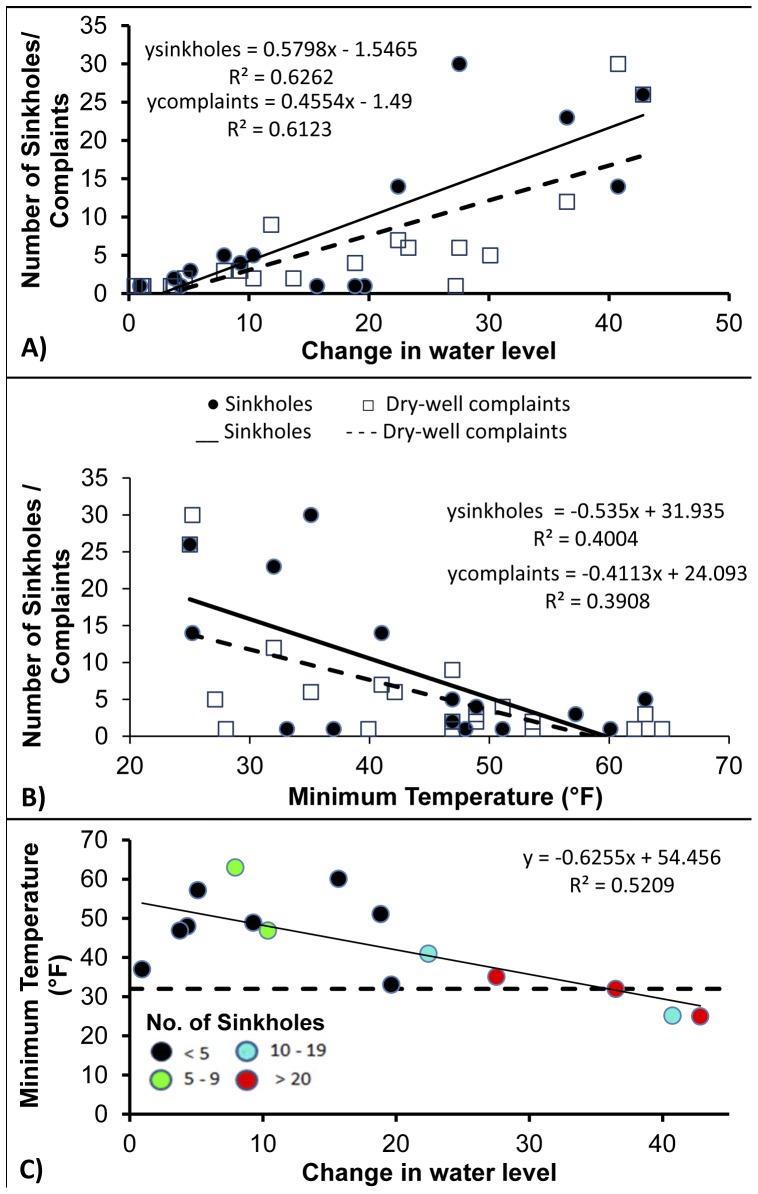
Graph illustrating the relationship between dry-well complaints and sinkholes during 2010 in relation to (A) water level change (ft), (B) minimum temperature (°F) and (C) the relationship between minimum temperature and changes in water level to the number of sinkholes.

A strong positive correlation was found between the occurrence of dry well complaints (*n = 22, r = 0.783, P>0.05*)/sinkholes (*n = 15, r = 0.791, P>0.05*) and change in water level (Figure 4A) and a strong negative correlation between sinkholes (*n = 15, r = 0.632, P>0.05*)/dry well complaints (*n = 22, r = 0.625, P>0.05*) and minimum temperature (Figure 4B). Furthermore, when temperatures fall below 41°F and water levels fall more than 20 ft, the number of sinkholes increase greatly (*N* >10) (Figure 4C).

### Economic Impact

For 2007, the total cost associated for dry well complaints was $12,859 (*N* = 5) and $472,951 (*N* = 124) during 2010. In 2010, the total estimated cost of using freeze-cloth to protect strawberry plants was estimated to be $16.5 million for a total of 6,900 acres of strawberry fields. Since the cost of using the freeze-cloth is subsided, the estimated cost to the farmers would have been $4.1 million, while the total estimated cost to SWFWMD would have been $12.4 million.

## Discussion

The occurrence of sinkholes as a result of groundwater pumping is not limited to Florida. Subsidence has also been documented in areas where increasing quantities of water are removed for agricultural and industrial use in other parts of USA [43], [44], particularly California (e.g. Santa Clara Valley [45] and San Joaquin Valley [46]), and regions of China (e.g. Su-Xi-Chang [47]). Although we only investigated how water-based crop protection methods impact the area surrounding strawberry farms in this study, there are a number of other agricultural systems in Florida that also use this method to protect crops. These include, citrus, fern, vegetable, vegetation, nursery and ornamental farms and tropical fish farms [9], [48], [49]. Of these, strawberries use the most water (16,200 gallons/acre vs. 2,100 (citrus) to 13,500 (Ferns) [48]. Many farmers use the Florida Automated Weather Network (FAWN) Data, an internet based tool, to plan for frost-freeze events and schedule irrigation times [48]. Even efficient tools such as FAWN, which are useful in reducing water depletion, can only help so much since it is the quick water withdrawal rates that can be damaging and cause dry wells and development of sinkholes [50]. Since Florida is mainly composed of limestone [17], it is likely that sinkhole and dry well complaints will continue to develop in agricultural areas where extreme pumping of water occurs and warrants future investigations to determine if the pattern exhibited in strawberry growing regions is also likely to occur in other agronomic cropping areas during frost-freeze events.

In this study, we found that the majority of the sinkhole and dry well complaints occurred within 1/4 mile of strawberry fields. A strong correlation was not only found between minimum temperature and change in water level but also between the occurrence of sinkholes and dry well complaints with ground water pumping during the 2010 frost-freeze event. The results from this study further support the findings of previous studies [14], [23], [30]. The main difference and improvement between the aforementioned studies and this study is that they each analyzed a single event for a single year. This study not only analyzed 2 frost-freeze events but compared those to a non-frost-freeze year as well as examined the incidence of sinkholes and dry well complaints over a 25 year time period. In addition, we were able to show that the location of dry well complaints as well as location of permits where more than 1 MGPD of water can be extracted (Figure 2C) were in close proximity to strawberry farms. This study benefited from improved data collection since 1985 as well as the use of GIS, which were not utilized previously. Furthermore, GIS allowed for the visualization, examination and modeling of data to assist in understanding the occurrence between sinkholes/dry-wells complaints in relation to strawberry farms, water level/change and minimum temperatures.

Throughout the time scale of this study, Florida has experienced several droughts that include: early 1980’s, 1989–1990, 1999–2001, 2006–2007 [51]. These have had considerable impact on water levels in this area, resulting in the need for additional water supplies. Until 2002, the main source of water was supplied from groundwater sources after which alternative sources, such as desalinated water and surface water, have been used [52]. During 2001, a large number of dry well complaints were recorded. There is a strong possibility that these may be as a result of the drought conditions and reliance on groundwater sources.

After the January 2010 frost-freeze event, SWFWMD adopted a comprehensive management plan to address future freeze events and help reduce future effects of sinkholes, potentially caused by irrigation [13]. The management plan includes several recommendations, the relevant points will be discussed based on the findings from this study and include; (i) establishment of critical minimum water level (ii) install automatic meter reading devices; (iii) ensure more consistent and reliable data recording; (iv) expand data collection network for freeze events; and (v) increase incentives for using alternative frost-freeze protection methods.

- Establishment of critical minimum water level: The research findings from this study indicate that during the 2010 frost-freeze event when water levels dropped below 20 ft the number of sinkholes increased substantially. Therefore, establishing a critical minimum water level would be useful in eliminating the drastic water level drops to the aquifer as seen during 2010 when water levels dropped approximately 60 ft in seven days. Since the freeze of 2010, specific rules addressing the Dover/Plant City WUCA (Water Use Caution Area), MAL (Minimum Aquifer Level), and MALPZ (Minimum Aquifer Level Protection Zone) have been implemented by SWFWMD (see [49] details).
- Install automatic meter reading devices; Farmers are currently permitted to extract a certain amount of water, but most of these pumps currently do not report accurate daily production quantities. Automatic meter reading devices will assist in identifying fields where more ground water is being pumped than permitted (Figure 2C). The data recorded from these meters can help improve our understanding of the aquifer and fine-tune the critical minimum water levels that have been set (see (i)) particularly during years when water levels are below normal.
- Ensure more consistent and reliable data recording protocols to track reported sinkhole and dry well complaints to SWFWMD. During this study 688 dry well complaints were omitted from the temporal analysis because the dates of when they occurred were not recorded. The addition of an “Event Date” attribute field to the storage database to track the date a sinkhole or dry well complaint incident is reported would allow for better spatial and temporal analysis of events.
- Expand the data collection network for freeze events to include additional temperature and water level monitoring sites would allow for improved monitoring. Consistent and reliable data recording was a problem throughout this study. Initial analysis of the water level data found that over the 25 years the most consistent records for temperature data was at the Tampa International Airport location, 23 miles east of the study site. Data for the sites closer to the strawberry farms only contained data for the past 16 years Since a strong correlation was found between the occurrence of sinkholes and dry well complaints with changes in water level (and minimum temperature and changes in water levels) improving monitoring of these factors will only enhance forecasting capabilities and future recommendations. The installation of a number of additional monitoring wells in the Dover/Plant City area has recently been completed to improve the understanding of freeze protection drawdown characteristics and ultimately improve groundwater flow modeling, as illustrated by [49]. In addition, these data can be used to predict density of sinkholes as shown by Doctor *et al.* [53]; identify criteria for minimizing sinkhole risk [54]; and map irrigation requirements [55].
- Finally, a key recommendation from the management plan was to increase incentives for using alternative frost-freeze protection methods so as to alleviate the use of excessive water usage in the Dover/Plant City Area. One alternative that was examined was the use of frost-freeze cloth. Our analysis showed that the current cost of implementing this method is prohibitive when compared with the cost of repairing dry wells. Although, incentives and cooperation systems, such as FARMS, could be key in reducing groundwater pumping, to make this a viable alternative the cost of the cloth would need to be reduced substantially. However, an advantage of utilizing this method is that the cloth can be re-used and therefore both the farmers and SWFWMD would both be faced with a high initial cost which would be substantially reduced over time. Future cost to the farmer would be the cost of labor to place the cloth over the crops.

For this study, we only analyzed the cost of repairing dry well complaints. The cost of repairing damages caused by sinkholes was not included since we were analyzing the cost incurred by the water authorities. However, additional costs of damages caused by the sinkholes were estimated to be much higher during 2010 (e.g. $7.6 million [56]) and represented in this analysis.

In conclusion, frost-freeze events will continue to impact the environment in Florida if water-based methods are used to protect crops. A strong correlation between minimum temperature and water level/change in water level was found during the 2010 frost-freeze event. The occurrence of sinkholes is likely to increase when minimum temperatures fall below 41°F and water levels drop by more than 20 ft. In addition, prolonged droughts are likely to affect the number of dry well complaints and sinkholes. Years where base water levels are already lower than normal, combined with cold temperatures are likely to lead to the development of numerous sinkholes and dry well complaints as recorded during the winter of 2010/2011.

## Supporting Information

Figure S1 **Total number of sinkholes reported to the Geological Survey in Florida by decade between 1950 and 2010 (FGS, 2011).**
(TIF)
