# Supplementary figures and images for: A GIS Analysis of the Relationship between Sinkholes, Dry-Well Complaints and Groundwater Pumping for Frost-Freeze Protection of Winter Strawberry Production in Florida
Source: PLoS One. 2013 Jan 11;8(1):e53832. doi: 10.1371/journal.pone.0053832 (PMC3543258; doi:10.1371/journal.pone.0053832)

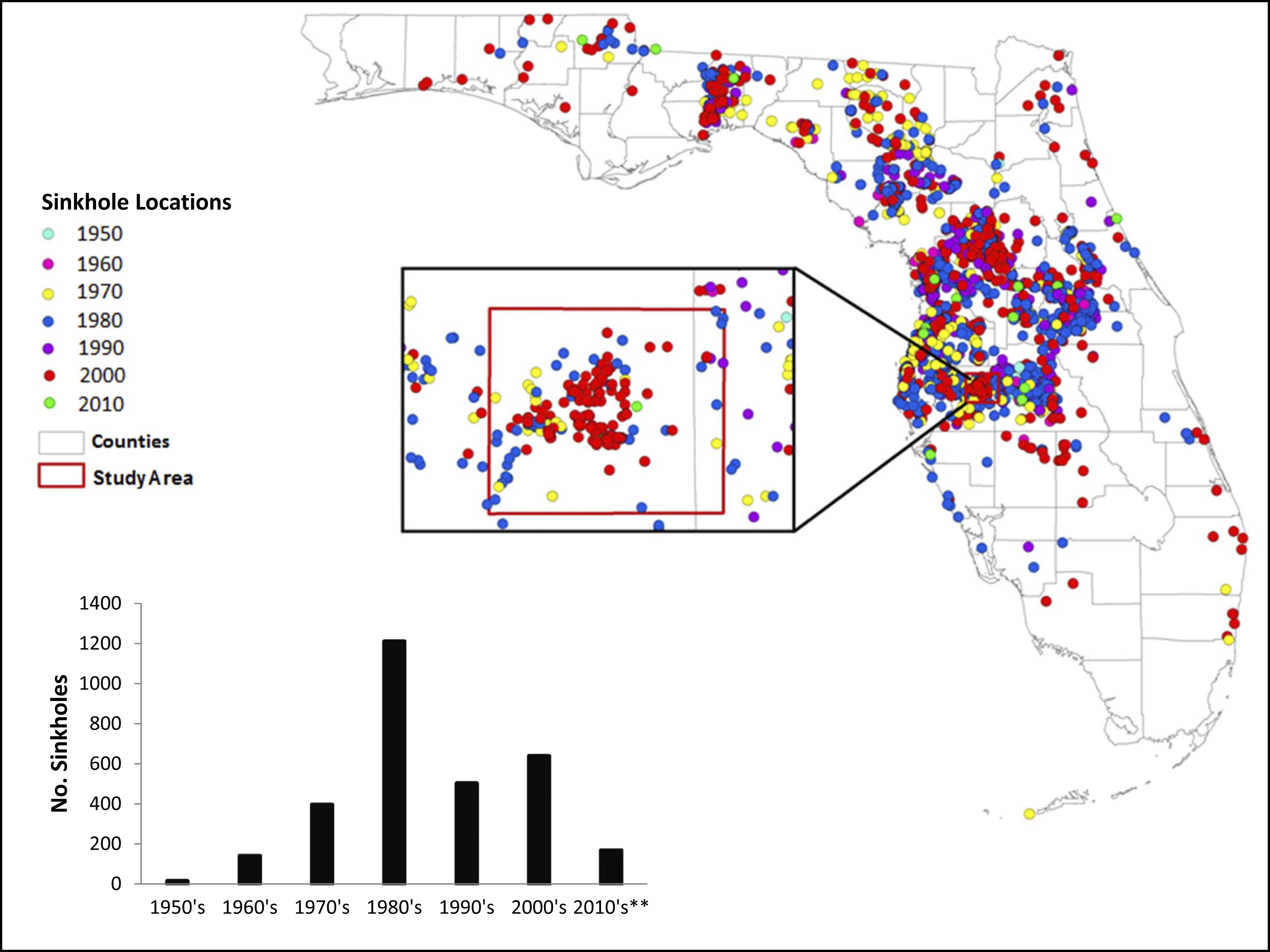

Supplement: Figure S1 — Total number of sinkholes reported to the Geological Survey in Florida by decade between 1950 and 2010 (FGS, 2011). (TIF) [file pone.0053832.s001.tif]
